# Effects of a long-term lifestyle modification programme on peripheral neuropathy in overweight or obese adults with type 2 diabetes: the Look AHEAD study

**DOI:** 10.1007/s00125-017-4253-z

**Published:** 2017-03-27

**Authors:** The Look AHEAD Research Group

**Affiliations:** c/o E. S. Horton, Joslin Diabetes Center, One Joslin Place, Boston, MA 02215 USA

**Keywords:** Clinical diabetes, Neuropathy-somatic, Weight regulation and obesity

## Abstract

**Aims/hypothesis:**

The aim of this study was to evaluate the effects on diabetic peripheral neuropathy (DPN) of a long-term intensive lifestyle intervention (ILI) programme designed to achieve and maintain weight loss.

**Methods:**

Beginning in 2001, a total of 5145 overweight or obese people with type 2 diabetes, aged 45–76 years, participating in the multicentre Look AHEAD (Action for Health in Diabetes) study were randomised to ILI (*n* = 2570) or to a diabetes support and education (DSE) control group (*n* = 2575) using a web-based management system at the study coordinating centre at Wake Forest School of Medicine (Winston-Salem, NC, USA). Randomisation was stratified by clinical centre and was not revealed to the clinical staff responsible for obtaining data on study outcomes. Because of the nature of the study, patients and the local centre interventionists were not blinded to the study group assignments. In addition, the coordinating centre staff members responsible for data management and statistical analyses were not blinded to the study group assignments. The interventions were terminated in September 2012, 9–11 years after randomisation, but both groups continued to be followed for both primary and secondary outcomes. Neuropathy evaluations included the Michigan Neuropathy Screening Instrument (MNSI) questionnaire completed at baseline in 5145 participants (ILI *n* = 2570, DSE *n* = 2575) and repeated annually thereafter and the MNSI physical examination and light touch sensation testing conducted in 3775 participants (ILI *n* = 1905, DSE *n* = 1870) 1–2.3 years after discontinuation of the intervention.

**Results:**

At baseline, the MNSI questionnaire scores were 1.9 ± 0.04 and 1.8 ± 0.04 in the ILI and DSE groups, respectively (difference not statistically significant). After 1 year, when weight loss was maximal in the ILI group (8.6 ± 6.9%) compared with DSE (0.7 ± 4.8%), the respective MNSI scores were 1.7 ± 0.04 and 2.0 ± 0.04 (*p* ≤ 0.001). Subsequently, the scores increased gradually in both groups, but remained significantly lower in the ILI group for the first 3 years and at the end of follow-up. In both groups, there was a significant association between changes in the MNSI scores and changes in body weight, HbA_1c_ and serum lipids. There were no significant between-group differences in the proportions of participants with MNSI physical examination scores ≥2.5, considered to be indicative of diabetic neuropathy. The light touch sensation measured separately in either the right or left big toes (halluces) did not differ between ILI and DSE, but when the data were combined for both toes, light touch was better preserved in the ILI group.

**Conclusions/interpretation:**

ILI resulted in a significant decrease in questionnaire-based DPN, which was associated with the magnitude of weight loss. In both the ILI and DSE groups, changes in the MNSI score were also related to changes in HbA_1c_ and lipids. There were no significant effects of ILI on physical examination measures of DPN conducted 1–2.3 years after termination of the active intervention, except for light touch sensation, which was significantly better in the ILI group when measurements were combined for both toes. However, a potential limiting factor to the interpretation of the physical examination data is that no baseline studies are available for comparison.

**Trial registration:**

ClinicalTrials.gov NCT00017953.

**Funding:**

This work was funded by the National Institutes of Health through cooperative agreements with the National Institute of Diabetes and Digestive and Kidney Diseases.

**Electronic supplementary material:**

The online version of this article (doi:10.1007/s00125-017-4253-z) contains peer-reviewed but unedited supplementary material, which is available to authorised users.

## Introduction

Diabetic neuropathies are a heterogeneous group of disorders of varying aetiology and clinical presentation [1]. The most common form is symmetrical diabetic peripheral neuropathy (DPN), which mainly affects the lower extremities and is a major cause of morbidity because of its effects on risk for subsequent ulcers, amputation and disability. DPN is associated with long-standing hyperglycaemia and its associated metabolic abnormalities, such as increased polyol flux, formation of advanced glycation end-products and increased oxidative stress, and with dyslipidaemia and other cardiometabolic risk factors [2, 3]. It is a common complication in people with type 2 diabetes and its response to improved glucose control may not always be as good as that seen in people with type 1 diabetes [4]. Several classes of medication have been shown to be effective in the management of painful DPN, including tricyclic antidepressants (amitriptyline, nortriptyline, imipramine), serotonin–noradrenaline (norepinephrine) reuptake inhibitors (duloxetine, venlafaxine) and voltage-gated calcium channel ligands (gabapentin, pregabalin) [5], but these agents have not been shown to prevent or alter the long-term progression of DPN. The effects of a programme of intensive lifestyle modification (ILI) focusing on weight reduction and increased physical activity on the development and or progression of DPN in people with type 2 diabetes have not been well studied, although a lifestyle programme in people with impaired glucose tolerance was shown to improve intraepidermal nerve fibre density [6].

The Look AHEAD (Action for Health in Diabetes) Study (ClinicalTrials.gov registration no. NCT00017953), was a multicentre, randomised clinical trial that compared the effects of an ILI programme with a control group receiving diabetes support and education (DSE) on the development of cardiovascular disease [7, 8]. The ILI focused on weight loss and physical activity. We have now examined the effects of the ILI and DSE programmes on the development and/or progression of DPN over a period of 11–12 years. To measure DPN, we used both a subjective measure, the Michigan Neuropathy Screening Instrument (MNSI) questionnaire, and objective data from the MNSI physical examination and Semmes–Weinstein 10 g monofilament examination (monofilament exam) for light touch sensation in the feet [9–16].

The main hypothesis was that the ILI programme would decrease the development and/or progression of DPN over the course of this long-term study.

## Methods

### Study design and participants

In the Look AHEAD study, overweight or obese individuals with type 2 diabetes mellitus, age 45–76 years, were randomly assigned 1:1 to ILI or DSE at 16 clinical centres in the USA. The design, power calculations, methods, baseline characteristics and main results have been described previously [7, 8]. Exclusion criteria included, but were not limited to, the inability to walk two blocks, a history of non-traumatic leg amputation or the presence of a significant abnormality on a maximum exercise stress test. Thus, some potential participants with clinically significant diabetic neuropathy may have been excluded from the study prior to randomisation.

In September 2012, 9–11 years after the initial enrolment and randomisation of participants, the active intervention phase of the trial was stopped by the trial’s sponsor based on the recommendation of the Data Safety Monitoring Board after a futility analysis indicated that the primary study endpoint, the post-randomisation occurrence of one of a composite of cardiovascular events or cardiovascular death, would not be met. The study design was converted to a long-term observation trial entitled the Look AHEAD Continuation Study (Look AHEAD-C). The MNSI physical examination and the monofilament exam were performed 1–2.3 years (mean 1.6 years) after discontinuation of the active intervention phase of the study.

The participating centres each received local institutional human subjects review board approval and written informed consent was obtained from each participant prior to enrolment.

### Randomisation and masking

Participants in the Look AHEAD trial were randomised in 2001–2004 using a web-based management system at the study coordinating centre at Wake Forest School of Medicine (Winston-Salem, NC, USA). Randomisation was stratified by clinical centre and was not revealed to the clinical staff responsible for obtaining data on study outcomes. However, because of the nature of the study, patients and the local centre interventionists were not blinded to the study group assignments. In addition, the coordinating centre staff members responsible for data management and statistical analyses were not blinded to the study group assignments.

### The MNSI

The MNSI questionnaire is a self-administered questionnaire that was completed at baseline and annually throughout the study. It consists of 15 questions, 13 of which have a positive response scored as 1 point and two of which have a negative response scored as 1 point, giving a possible maximum total of 15 points [9] (see below). A score of ≥4 was considered abnormal, based on the analysis of its sensitivity and specificity in identifying DPN, and this was confirmed by nerve conduction testing in participants in the Diabetes Control and Complications Trial/Epidemiology of Diabetes Interventions and Complications (DCCT/EDIC) trial [13]. The presence of a lower extremity amputation (question 15) was confirmed by physical examination and review of serious adverse event reports throughout the study.
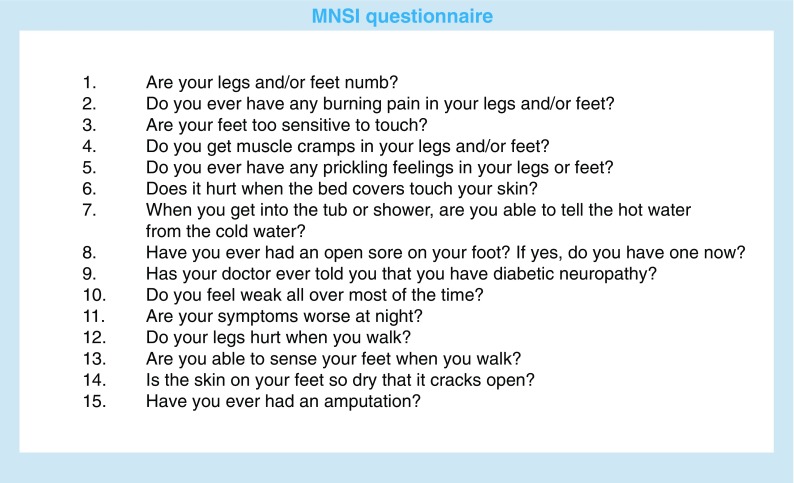


The MNSI physical examination was performed once, 11–12 years after randomisation and 1–2.3 years after discontinuation of the active intervention. The criteria for scoring this test were as follows: (1) inspection of each foot for deformities, dry skin, calluses, infections and fissures (the presence of any of these abnormalities on a foot was scored as 1 point); (2) each foot was also inspected for ulcers and each foot with an ulcer received a score of 1 point; (3) ankle reflexes were tested and scored as normal (0 points), present only with reinforcement (0.5 points) and totally absent (1 point); (4) vibration sense was tested bilaterally on the dorsum of the big toe using a 128 Hz tuning fork and compared with the duration of the vibration detected on the examiner’s distal forefinger. If the examiner detected the vibration for <10 s longer than the participant the vibration sense was normal (0 points), if it was ≥10 s longer, vibration sense was reduced (0.5 points) and if not felt at all by the participant it was absent (1 point). The maximum score for the MNSI physical examination was 4 points for each foot, giving a total maximum score of 8 points [9]. In the case of a unilateral foot amputation, the score for the remaining foot was doubled. A score of ≥2.5 was considered abnormal based on the studies of Lunetta et al [10] and its use in evaluating the MNSI physical examination results in individuals with type 1 diabetes in the DCTC/EDIC trial [13].

### Sensory nerve function testing

For the assessment of light touch sensation, a Semmes–Weinstein 10 g monofilament was used. Ten touches were administered to the dorsal surface of the big toe between the base of the nail and the proximal interphalangeal joint on both the right and left foot. In each location, perception of 8–10 touches was scored as normal (0 points), 1–7 touches as reduced (0.5 points) and 0 touches as absent (1 point) [12]. In an additional post hoc analysis, if a participant perceived <8 touches on either big toe they were considered to have at least some minimal evidence of decreased light touch sensation in the feet.

### Statistical analyses

All data collected since randomisation were included in this analysis. A linear mixed-effects model was used to analyse the MNSI questionnaire score. The model included an indicator variable for intervention assignment, follow-up time and the interaction between the intervention and follow-up time while adjusting for the baseline MNSI score. Least square means and the corresponding 95% CIs were estimated and plotted to portray the trend over time. The average post-randomisation levels of the overall MNSI score for DSE and ILI were estimated and compared using contrasts.

As a secondary analysis, we examined the association between various risk factors and mean MNSI scores. Specifically, we used weight change from baseline and systolic blood pressure (SBP) measured annually during the Look AHEAD-C study. We also used HbA_1c_, LDL-cholesterol, HDL-cholesterol, triacylglycerol and triacylglycerol:HDL-cholesterol ratio measured annually up to and including year 4 and then biannually through the remainder of Look AHEAD. An additional measure of HbA_1c_ in the Look AHEAD-C study was included in analyses as well. All these risk factors were modelled as time-varying predictors. Each risk factor and its interaction with the intervention assignment were added to the model separately.

The presence of MNSI questionnaire scores ≥4 and a participant’s responses to each individual MNSI item were analysed using logistic regression models appropriate for repeated binary outcomes. The models used generalised estimating equations (GEE), a logit link and a binomial variance and included baseline outcome measure, an indicator variable for intervention assignment, follow-up time and the interaction between the intervention and follow-up time. Overall OR and 95% CIs for ILI compared with DSE were constructed from the fitted models.

The MNSI physical examination and the monofilament test were performed once in the Look AHEAD-C study, 1–2.3 years after discontinuation of the active intervention phase of the study. The presence of MNSI physical examination scores ≥2.5 was compared between DSE and ILI using a *χ* ^2^ test. Abnormal monofilament test results in either foot were analysed in a similar fashion.

Finally, we examined medication use that was potentially related to DPN. The use of any biguanides was summarised for each year and each intervention group. It was also modelled as a time-varying predictor to assess its association with MNSI questionnaire scores. The use of any medications to treat painful DPN (tricyclic antidepressants, serotonin and noradrenaline reuptake inhibitors and voltage-gated calcium channel ligands) was also summarised by year and by intervention group. All analyses were conducted using SAS 9.4 software (SAS, Cary, NC, USA). A two-sided *p* value of less than 0.05 was considered statistically significant.

## Results

### MNSI questionnaire results

**Fig. 1:**
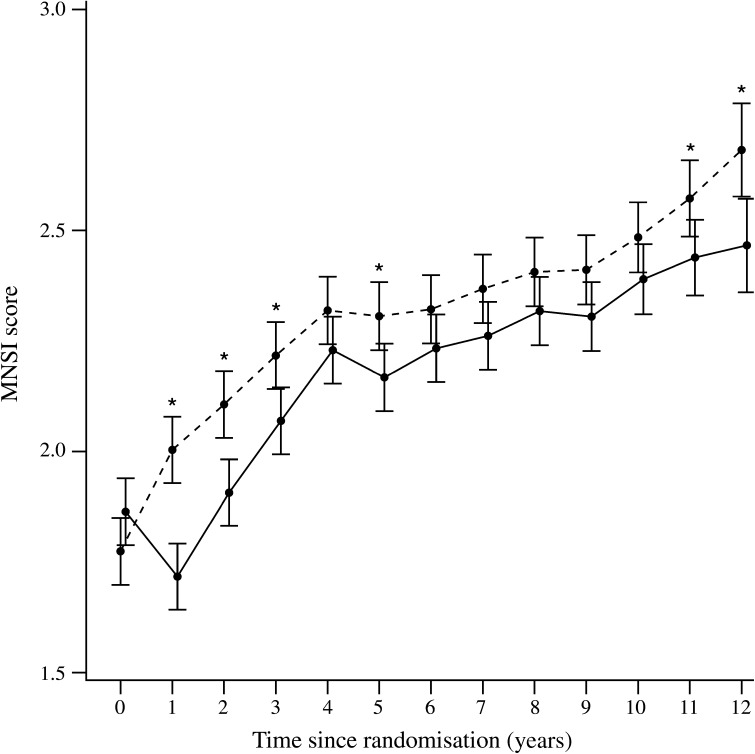
MNSI questionnaire scores at baseline and yearly in the DSE (dashed line) and ILI (solid line) groups. *p < 0.05

**Fig. 2:**
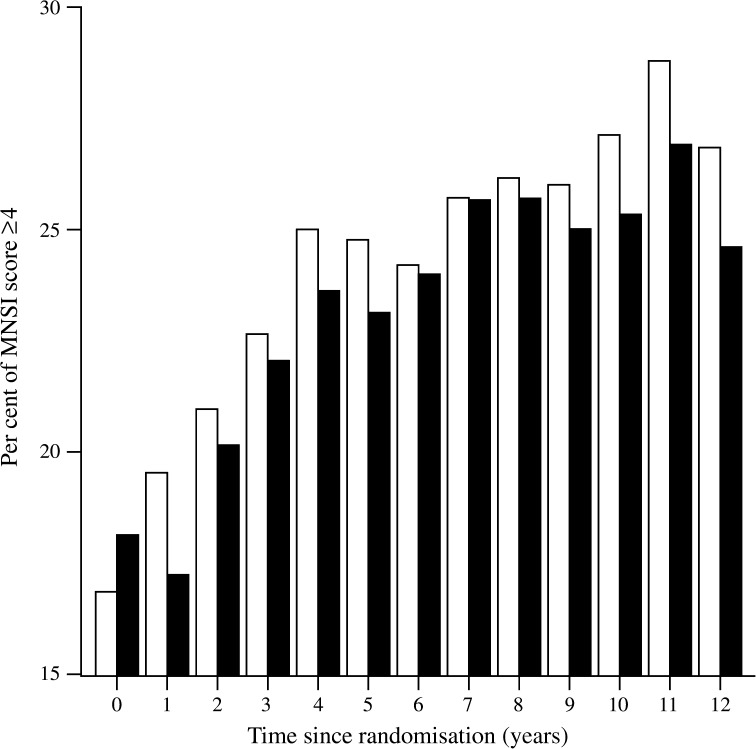
Percentage of MNSI questionnaire scores ≥4 in the DSE (white bars) and ILI (black bars) groups

At baseline, there was no significant difference in the MNSI questionnaire score between the ILI (*n* = 2570) and DSE groups (*n* = 2575), with mean scores of 1.9 ± 0.04 and 1.8 ± 0.04, respectively. However, at 1 year, when the ILI group had achieved maximum weight loss (8.6 ± 6.9% vs 0.7 ± 4.8% in the DSE group [17]), the MNSI questionnaire score had decreased to 1.7 ± 0.04 and was significantly lower than the DSE group score of 2.0 ± 0.04 (*p* < 0.001). Subsequently, the MNSI scores for both groups increased progressively over time, but remained significantly lower in the ILI group than in the DSE group for the first 3 years and in the final years of the follow-up (Fig. 1). The MNSI score averaged over the course of the study was 2.35 ± 0.03 and 2.21 ± 0.03 for the DSE and ILI group, respectively (*p* < 0.001). The percentage of participants with an MNSI questionnaire score ≥4 in each group is shown in Fig. 2. This percentage decreased in the ILI group at the end of the first year of active intervention and then increased gradually over the next several years to approximately 25%. In the DSE group there was a gradual increase in the percentage of participants with a score of ≥4 to approximately 27%. Over the entire course of the study, the average percentage of participants with an MNSI score of ≥4 was significantly lower in the ILI group than in the DSE group (OR [95% CI] 0.89 [0.81, 0.99], *p* = 0.026).

Compared with the DSE group, the ILI group had significantly fewer positive responses to questions 2, 4, 5, 10 and 12 on the questionnaire, namely less burning pain in the feet and legs, fewer muscle cramps, less prickling sensation in the legs and feet, less feeling weak all over and less leg pain when walking. There were no significant differences between the two groups in their responses to the other MNSI questions.

In both the DSE and ILI groups there was a significant association between the mean MNSI scores over the follow-up period and the mean weight change from baseline and the mean HbA_1c_ levels measured throughout the follow-up phase of the study. For each 1 kg decrease in weight from baseline there was a decrease in the MNSI score of 0.02 (*p* < 0.001) for both the DSE and ILI groups and for each increase in HbA_1c_ of 1% (NGSP units) there was an increase in the MNSI score of 0.04 (*p* < 0.001) in the DSE group and 0.06 (*p* < 0.001) in the ILI group. The mean MNSI scores were also associated negatively with HDL-cholesterol and positively with serum triacylglycerols and the triacylglycerol:HDL-cholesterol ratios. For each 0.259 mmol/l (10 mg/dl) decrease in HDL-cholesterol the MNSI score increased by 0.07 (*p* < 0.001) in the DSE group and by 0.05 (*p* = 0.0016) in the ILI group. For each 0.113 mmol/l (10 mg/dl) increase in triacylglycerol level there was an increase in the MNSI score of 0.006 (*p* < 0.001) in the DSE group and 0.005 (*p* < 0.001) in the ILI group. For each unit increase in the triacylglycerol:HDL-cholesterol ratio the MNSI score increased by 0.02 (*p* < 0.001) in both the DSE and ILI groups. There was no significant association between the MNSI score and LDL-cholesterol or SBP in the DSE and ILI groups. There was no statistically significant interaction with the treatment for any of these risk factors.

### MNSI physical examination and sensory nerve testing results

The results of the cross-sectional MNSI physical examination and sensory nerve testing conducted in 3775 participants (ILI *n* = 1905, DSE *n* = 1870) 11–12 years after randomisation and 1–2.3 years after discontinuation of the active intervention phase of the study are shown in Table 1. A physical examination score of ≥2.5, indicative of the presence of some degree of peripheral neuropathy, was present in 67.8% of the participants in the DSE group and 67.4% of the participants in the ILI group (difference not statistically significant). The light touch sensation, measured by monofilament testing on both big toes and analysed separately, was abnormal (<8/10 pricks detected) in 28.3% of the participants in the DSE group and 25.6% of the participants in the ILI group on the right foot (difference not statistically significant) and 27.4% vs 24.4% (difference not statistically significant) of the participants, respectively, on the left foot. In an additional post hoc analysis the data were analysed as a reading of <8 perceived pricks on either the right or the left foot. This was present in 34.5% of the participants in the DSE group and 30.4% of the participants in the ILI group (*p* = 0.008), suggesting that the ILI group participants may have maintained slightly better light touch sensation in the feet than did the DSE group participants.

**Table 1:** MNSI and Semmes–Weinstein monofilament examination results

Test result	All participants (*n* = 3775)	DSE group (*n* = 1870)	ILI group (*n* = 1905)	*p* value for ILI vs DSE
MNSI physical examination score^a^				
MNSI score <2.5	1224 (32.42)	603 (32.25)	621 (32.60)	0.82
MNSI score ≥2.5	2551 (67.58)	1267 (67.75)	1284 (67.40)	
Monofilament testing of light touch perception^b^				
Left big toe 8–10	2790 (74.08)	1355 (72.58)	1435 (75.57)	0.11
Left big toe 1–7	793 (21.06)	414 (22.17)	379 (19.96)	
Left big toe 0	183 (4.86)	98 (5.25)	85 (4.48)	
Right big toe 8–10	2752 (73.06)	1338 (71.70)	1414 (74.38)	0.18
Right big toe 1–7	831 (22.06)	433 (23.20)	398 (20.94)	
Right big toe 0	184 (4.88)	95 (5.09)	89 (4.68)	
Either big toe <8	1222 (32.44)	644 (34.49)	578 (30.42)	0.008

Data are expressed as means (%)

^a^A score of ≥2.5 out of 8 is considered positive for neuropathy

^b^The perception of 8–10 touches is considered to be normal, 1–7 is decreased and 0 is absent light touch sensation

### Use of biguanides and medications for DPN

Additional analyses were conducted to determine whether there was an association between the use of biguanides and the MNSI questionnaire scores in either the ILI group or the DSE group and whether there were significant differences in the use of medications for treating painful DPN between the groups. Biguanides were used by 62% of the ILI participants and 61% of the DSE participants at baseline; at year 12, the respective percentage was 64% and 67%. Average use during the course of the study was slightly greater in the DSE group than in the ILI group. However, there was no association between the use of biguanides and the MNSI questionnaire scores. The use of medications to treat painful DPN, including tricyclic antidepressants, serotonin and noradrenaline reuptake inhibitors and voltage-gated calcium channel ligands was relatively low, being 4.2% of the ILI and 4.5% of the DSE participants at baseline, increasing to 13.6% and 14.7% of ILI and DSE participants, respectively, at year 12.

## Discussion

In the Look AHEAD study, a large, multicentre trial to determine the effects of an intensive lifestyle modification programme on the development of cardiovascular disease in overweight or obese people with type 2 diabetes, the impact of the lifestyle intervention programme (ILI group) on the development and progression of DPN was examined and compared with that in the randomised control group (DSE group). A combination of the MNSI questionnaire administered annually and a neurological examination conducted during the Look AHEAD-C phase of the study 11 or 12 years after randomisation of participants and 1–2.3 years after discontinuation of the active intervention phase of the study was used. The key findings were that the ILI intervention group had a significant decrease in the score on the MNSI questionnaire at the end of the first year of the study when maximal weight loss was achieved. Following this initial improvement, there was a progressive increase in the MNSI scores in both the ILI and DSE groups, but the ILI group maintained lower scores than the DSE group throughout the duration of the study. This is consistent with an initial and persistent beneficial effect of the ILI programme on symptoms commonly associated with DPN. Specific areas of benefit included less leg pain when walking, less leg weakness, fewer muscle cramps and less painful sensory neuropathy in the legs and feet.

When the neurological physical examinations were completed during the Look AHEAD-C phase of the study, approximately two-thirds of the participants had some evidence of DPN and there was no significant difference between the ILI and DSE groups. In addition, approximately 27–33% of the participants had mild to severe loss of monofilament light touch sensation. When tested individually on the right or left big toe, no significant differences were seen between the DSE and ILI groups. However, when analysed as decreased or absent light touch sensation in either the right or left foot, significantly fewer people in the ILI group than in the DSE group had evidence of at least some loss of sensation.

In analyses of additional factors that might influence the presence or severity of DPN in the study participants, we determined that the use of biguanides was similar in the ILI and DSE groups and was not associated with the MNSI questionnaire scores. However, no data were collected on vitamin B_12_ levels during the study to evaluate the possibility that the use of biguanides might be associated with significant vitamin B_12_ deficiency, which could contribute to the development of peripheral neuropathy. The use of medications to treat painful DPN was relatively low and not different between the two groups. Thus, it seems unlikely that the use of these medications would have significantly affected the study results.

Taken together, these findings indicate that an intervention focused on weight loss and increased physical exercise in overweight people with type 2 diabetes may reduce the clinical manifestations of DPN initially and that this beneficial effect can be maintained for many years. Whether this is associated primarily with the achievement or maintenance of weight loss, changes in physical activity, better glycaemic control or improvements in blood pressure or lipids, or is due to other factors, is not clear. The significant positive associations of the MNSI scores with HbA_1c_ and change in body weight and the weaker, but statistically significant, associations with serum HDL-cholesterol, triacylglycerols and the triacylglycerol:HDL-cholesterol ratio in both the DSE and ILI groups support the importance of these risk factors in the development of DPN and their amelioration by the lifestyle intervention programme. The associations between LDL-cholesterol or SBP and the MNSI scores were either not significant or very weak in the two treatment groups, suggesting that these factors did not play a clinically significant role in the development of DPN in the Look AHEAD participants.

Several limitations should be noted. Since the study exclusion criteria eliminated participants who had a previous non-traumatic lower extremity amputation, could not walk for two blocks or who had a significantly abnormal exercise stress test, some volunteers with significant diabetic neuropathy may have been excluded from the study prior to randomisation, limiting the generalisability of our findings. We do not have baseline data for the MNSI physical examination or monofilament light touch testing to compare with the data collected during the Look AHEAD-C phase of the study. However, for those participants in the Look AHEAD study who were randomised to the ILI group programme, there was a significant decrease in the MNSI questionnaire scores during the initial phases of the study, when significant weight loss occurred, and this beneficial effect persisted for at least 11–12 years when compared with the MNSI scores in the DSE control group. However, in contrast to the MNSI questionnaire results, with the exception of slightly reduced loss of light touch sensation in the ILI group, there were no significant differences in the MNSI physical examination scores in either group. It should be recognised that, because of their limitations, the MNSI questionnaire and physical examination and the Semmes–Weinstein monofilament testing for light touch sensation may not be ideal for diagnosing diabetic neuropathy in the participants in this study and that the results have not been confirmed by measurement of nerve conduction velocity or by biopsy techniques. Unfortunately, the nature of this large, multicentre trial has precluded obtaining this type of information, so we have had to rely on the use of the MNSI questionnaire and physical examination methods for evaluating DPN that are commonly used in clinical practice.

### Electronic supplementary material

ESM (PDF 24 kb)

## Appendix

### Writing group

Edward S. Horton, Research Division, Joslin Diabetes Center, Boston, MA, USA

Haiying Chen, Department of Biostatistical Sciences,Wake Forest University Health Sciences,Winston-Salem, NC, USA

David M. Nathan, Diabetes Center, Massachusetts General Hospital, Boston, MA, USA

Xavier Pi-Sunyer, Division of Endocrinology, Department of Medicine, Columbia University Medical Center, New York, NY, USA

William C. Knowler, Diabetes Epidemiology and Clinical Research Section, National Institute of Diabetes and Digestive and Kidney Diseases, Phoenix, AZ, USA

Edward W. Gregg, Division of Diabetes Translation, Centers for Disease Control, Atlanta, GA, USA

Judy L. Bahnson, Department of Biostatistical Sciences,Wake Forest University Health Sciences,Winston-Salem, NC, USA

### Authors

Edward S. Horton, Research Division, Joslin Diabetes Center, Boston, MA, USA

Judy L. Bahnson, Department of Biostatistical Sciences,Wake Forest University Health Sciences,Winston-Salem, NC, USA

George Blackburn, Department of Surgery, Beth Israel Deaconess Medical Center, Boston, MA USA

George A. Bray, Clinical Obesity, Pennington Biomedical Research Center, Louisiana State University, Baton Rouge, LA, USA

Jeanne Charleston, Department of Epidemiology, Johns Hopkins Bloomberg School of Public Health, Baltimore, MD, USA

Haiying Chen, Department of Biostatistical Sciences,Wake Forest University Health Sciences,Winston-Salem, NC, USA

Jeanne M. Clark, Division of General Internal Medicine, Department of Medicine,The Johns Hopkins University School of Medicine, Baltimore, MD, USA

Mace Coday, Department of Preventive Medicine, University of Tennessee Health Science Center, Memphis, TN, USA

Jeffrey M. Curtis, Family Medicine, St Joseph’s Hospital and Medical Center, Phoenix, AZ, USA

Mary Evans, National Health Institute, National Institute of Diabetes and Digestive and Kidney Diseases, Bethesda, MD, USA

Michelle E. Fisher, Department of Psychiatry & Human Behavior, Warren Alpert Medical School of Brown University; Weight Control & Diabetes Research Center, The Miriam Hospital, Providence, RI, USA

John P. Foreyt, Department of Medicine, Baylor College of Medicine, Houston, TX, USA

Frank L. Greenway, Clinical Trials, Pennington Biomedical Research Center, Louisiana State University, Baton Rouge, LA, USA

Edward W. Gregg, Division of Diabetes Translation, Centers for Disease Control, Atlanta, GA, USA

Helen P. Hazuda, Department of Clinical Epidemiology, University of Texas Health Science Center, San Antonio, TX, USA

James O. Hill, Department of Clinical Nutrition, Anschutz Health & Wellness Center, University of Colorado, Aurora, CO, USA

John M. Jakicic, Department of Health and Physical Activity, University of Pittsburgh, Pittsburgh, PA, USA

Robert W. Jeffery, Department of Epidemiology & Community Health, University of Minnesota, Minneapolis, MN, USA

Karen C. Johnson, Department of Preventive Medicine, University of Tennessee Health Science Center, Memphis, TN, USA

Steven E. Kahn, Division of Metabolism, Endocrinology, and Nutrition, VA Puget Sound Health Care System and University of Washington, Seattle, WA, USA

William C. Knowler, Diabetes Epidemiology and Clinical Research Section, National Institute of Diabetes and Digestive and Kidney Diseases, Phoenix, AZ, USA

Mary Korytkowski, Division of Endocrinology and Metabolism, University of Pittsburgh, PA, USA

Anne Kure, VA Puget Sound Health Care System and University of Washington, Seattle, WA, USA

David Lefkowitz, Department of Neurology, Wake Forest University Health Sciences,Winston-Salem, NC, USA

Barbara J. Maschak-Carey**,** Center for Weight and Eating Disorders, University of Pennsylvania Philadelphia, PA, USA

Sara Michaels, Family Medicine, St Joseph’s Hospital and Medical Center, Phoenix, AZ, USA

Maria G. Montez, Department of Kinesiology, Health, and Nutrition, University of Texas Health Science Center, San Antonio, TX, USA

David M. Nathan, Diabetes Center, Massachusetts General Hospital, Boston, MA, USA

Jennifer Patricio, Division of Endocrinology, Department of Medicine, Columbia University Medical Center, New York, NY, USA

Anne Peters, Department of Clinical Medicine, Keck School of Medicine, Los Angeles, CA, USA

Xavier Pi-Sunyer, Division of Endocrinology, Department of Medicine, Columbia University Medical Center, New York, NY, USA

Henry Pownall, Department of Cardiology, Institute for Academic Medicine, Houston Methodist, Houston, TX, USA

Bruce Redmon, Department of Medicine, University of Minnesota Medical School, Minneapolis, MN, USA

Judith G. Regensteiner, Department of Medicine, University of Colorado School of Medicine, Aurora, CO, USA

Monika Safford, Department of Medicine, Weill Cornell Medicine, New York, NY, USA

Helmut Steinburg, Department of Endocrinology, University of Tennessee, Memphis, TN, USA

Thomas A. Wadden, Department of Psychiatry, University of Pennsylvania, Perelman School of Medicine, Philadelphia, PA, USA

Rena R. Wing, Department of Psychiatry & Human Behavior, Warren Alpert Medical School of Brown University, Weight Control & Diabetes Research Center, The Miriam Hospital, Providence, RI, USA

Ping Zhang, Division of Diabetes Translation, Centers for Disease Control, Atlanta, GA, USA

## Abbreviations

DCCT/EDIC: Diabetes Control and Complications Trial/Epidemiology of Diabetes Complications
DPN: Diabetic peripheral neuropathy
DSE: Diabetes support and education
ILI: Intensive lifestyle intervention
Look AHEAD: Action for Health in Diabetes
Look AHEAD-C: Look AHEAD Continuation
MNSI: Michigan Neuropathy Screening Instrument
SBP: Systolic blood pressure
