# Supplementary material for: Effects of a long-term lifestyle modification programme on peripheral neuropathy in overweight or obese adults with type 2 diabetes: the Look AHEAD study
Source: Diabetologia. 2017 Mar 27;60(6):980–8. doi: 10.1007/s00125-017-4253-z (PMC5423967; doi:10.1007/s00125-017-4253-z)
Supplement: Supplementary file 1 — (PDF 24 kb) [file 125_2017_4253_MOESM1_ESM.pdf]

Manuscript number: 16 1854.R1

Electronic supplementary materials

Clinical Sites:

The Johns Hopkins University Frederick L. Brancati, MD, MHS[1*]; Jeanne M. Clark, MD, MPH[1] (Co-Principal Investigators); Lee Swartz[2]; Jeanne Charleston, RN[3]; Lawrence Cheskin, MD[3]; Richard Rubin, PhD[3*]; Jean Arceci, RN; David Bolen; Danielle Diggins; Mia Johnson; Joyce Lambert; Sarah Longenecker; Kathy Michalski, RD; Dawn Jiggetts; Chanchai Sapun; Maria Sowers; Kathy Tyler

Pennington Biomedical Research Center George A. Bray, MD[1]; Allison Strate, RN[2]; Frank L. Greenway, MD[3]; Donna H. Ryan, MD[3]; Donald Williamson, PhD[3]; Timothy Church, MD[3]; Catherine Champagne, PhD, RD; Valerie Myers, PhD; Jennifer Arceneaux, RN; Kristi Rau; Michelle Begnaud, LDN, RD, CDE; Barbara Cerniauskas, LDN, RD, CDE; Crystal Duncan, LPN; Helen Guay, LDN, LPC, RD; Carolyn Johnson, LPN, Lisa Jones; Kim Landry; Missy Lingle; Jennifer Perault; Cindy Puckett; Marisa Smith; Lauren Cox; Monica Lockett, LPN

The University of Alabama at Birmingham Cora E. Lewis, MD, MSPH[1]; Sheikilya Thomas, PhD,MPH[2]; Monika Safford, MD[3]; Stephen Glasser, MD[3]; Vicki DiLillo, PhD[3]; Gareth Dutton, PhD, Charlotte Bragg, MS, RD, LD; Amy Dobelstein; Sara Hannum; Anne Hubbell, MS; Jane King, MLT; DeLavallade Lee; Andre Morgan; L. Christie Oden; Janet Wallace, MS; Cathy Roche, PhD, RN, BSN; Jackie Roche; Janet Turman

Harvard Center

Massachusetts General Hospital. David M. Nathan, MD[1]; Enrico Cagliero, MD[3]; Heather Turgeon, RN, BS, CDE[2]; Barbara Steiner, EdM; Valerie Goldman, MS, RDN[2]; Linda Delahanty, MS, RDN[3]; Ellen Anderson, MS, RDN[3]; Laurie Bissett, MS, RDN; Christine Stevens, RN; Mary Larkin, RN; Kristen Dalton, BS, Roshni Singh, BS

Joslin Diabetes Center: Edward S. Horton, MD[1]; Sharon D. Jackson, MS, RD, CDE[2]; Osama Hamdy, MD, PhD[3]; A. Enrique Caballero, MD[3]; Sarah Bain, BS; Elizabeth McKinney, BSN, RN; Barbara Fargnoli, MS,RD; Jeanne Spellman, BS, RD; Kari Galuski, RN; Ann Goebel-

Fabbri, PhD; Lori Lambert, MS, RD; Sarah Ledbury, MEd, RD; Maureen Malloy, BS; Kerry Ovalle, MS, RCEP, CDE

Beth Israel Deaconess Medical Center: George Blackburn, MD, PhD[1*]; Christos Mantzoros, MD, DSc[3]; Ann McNamara, RN

University of Colorado Anschutz Medical Campus James O. Hill, PhD[1]; Marsha Miller, MS RD[2]; Holly Wyatt, MD[3], Brent Van Dorsten, PhD[3]; Judith Regensteiner, PhD[3]; Debbie Bochert; Gina Claxton-Malloy RD Ligia Coelho, BS; Paulette Cohrs, RN, BSN; Susan Green; April Hamilton, BS, CCRC; Jere Hamilton, BA; Eugene Leshchinskiy; Loretta Rome, TRS; Terra Thompson, BA, Kirstie Craul, RD, CDE; Cecilia Wang, MD

Baylor College of Medicine John P. Foreyt, PhD[1]; Rebecca S. Reeves, DrPH, RD[2]; Molly Gee, MEd, RD[2]; Henry Pownall, PhD[3]; Ashok Balasubramanyam, MBBS[3]; Chu-Huang Chen, MD, PhD[3]; Peter Jones, MD[3]; Michele Burrington, RD, RN; Allyson Clark Gardner, MS, RD; Sharon Griggs; Michelle Hamilton; Veronica Holley; Sarah Lee; Sarah Lane Liscum, RN, MPH; Susan Cantu-Lumbreras; Julieta Palencia, RN; Jennifer Schmidt; Jayne Thomas, RD; Carolyn White; Charlyne Wright, RN; Monica Alvarez, PCT

The University of Tennessee Health Science Center

University of Tennessee East. Karen C. Johnson, MD, MPH[□]; Karen L. Wilson, BSN[□]; Mace Coday, PhD[3]; Beate Griffin, RN, BS; Donna Valenski; Polly Edwards; Brenda Fonda; Kim Ward

University of Tennessee Downtown. Helmut Steinburg, MD[3]; Carolyn Gresham, BSN[□]; Moana Mosby, RN; Debra Clark, LPN; Donna Green RN; Abbas E. Kitabchi, PhD, MD (retired)

University of Minnesota Robert W. Jeffery, PhD[1]; Tricia Skarphol, MA[2]; John P. Bantle, MD[3]; J. Bruce Redmon, MD[3]; Richard S. Crow, MD[3]; Scott J. Crow, MD[3]; Manami Bhattacharya, BS; Cindy Bjerk, MS, RD; Kerrin Brelje, MPH, RD; Carolyne Campbell; Mary Ann Forseth, BA; Melanie Jaeb, MPH, RD; Philip Lacher, BBA; Patti Laqua, BS, RD; Birgitta I. Rice, MS, RPh, CHES; Ann D. Tucker, BA; Mary Susan Voeller, BA

Columbia University Medical Center Xavier Pi-Sunyer, MD[1]; Jennifer Patricio, MS[2]; Carmen Pal, MD[3]; Lynn Allen, MD; Janet Crane, MA, RD, CDN; Lolline Chong, BS, RD; Diane Hirsch, RNC, MS, CDE; Mary Anne Holowaty, MS, CN; Michelle Horowitz, MS, RD; Raashi Mamtani, MS

University of Pennsylvania Thomas A. Wadden, PhD[1]; Barbara J. Maschak-Carey, MSN, CDE[2]; Robert I. Berkowitz, MD[3]; Gary Foster, PhD[3]; Henry Glick, PhD[3]; Shiriki Kumanyika, PhD RD, MPH[3]; Yuliis Bell, BA; Raymond Carvajal, PsyD; Helen Chomentowski; Renee Davenport; Lucy Faulconbridge, PhD;
Louise Hesson, MSN, CRNP; Sharon Leonard, RD; Monica Mullen, RD, MPH

University of Pittsburgh John M. Jakicic, PhD[1]; David E. Kelley, MD[1]; Jacqueline Wesche-Thobaben, RN, BSN, CDE[2]; Daniel Edmundowicz, MD[3]; Lin Ewing, PhD, RN[3]; Andrea Hergenroeder, PhD, PT, CCS[3]; Mary L. Klem, PhD, MLIS[3]; Mary Korytkowski, MD[3]; Andrea Kriska, PhD[3]; Lewis H. Kuller, MD, DrPH[3]; Amy D. Rickman, PhD, RD, LDN[3]; Rose Salata, MD[3]; Monica E. Yamamoto, DrPH, RD, FADA[3]; Janet Bonk, RN, MPH; Susan Copelli, BS, CTR; Rebecca Danchenko, BS; Tammy DeBruce, BA; Barbara Elnyczky; David O. Garcia, PhD; George A. Grove, MS; Patricia H. Harper, MS, RD, LDN; Susan Harrier, BS; Diane Heidingsfelder, MS, RD, CDE, LDN; Nicole L. Helbling, MS, RN; Diane Ives, MPH; Janet Krulia, RN, BSN, CDE; Juliet Mancino, MS, RD, CDE, LDN; Anne Mathews, PhD, RD, LDN; Lisa Martich, BS, RD, LDN; Meghan McGuire, MS; Tracey Y. Murray, BS; Anna Peluso, MS; Karen Quirin; Jennifer Rush, MPH; Joan R. Ritchea; Linda Semler, MS, RD, LDN; Karen Vujevich, RN-BC, MSN, CRNP; Kathy Williams, RN, MHA; Donna L. Wolf, PhD

The Miriam Hospital/Brown Medical School Rena R. Wing, PhD[1]; Renee Bright, MS[2]; Vincent Pera, MD[3]; Deborah Tate, PhD[3]; Amy Gorin, PhD[3]; Kara Gallagher, PhD[3]; Amy Bach, PhD; Barbara Bancroft, RN, MS; Anna Bertorelli, MBA, RD; Richard Carey, BS; Tatum Charron, BS; Heather Chenot, MS; Kimberley Chula-Maguire, MS; Pamela Coward, MS, RD; Lisa Cronkite, BS; Julie Currin, MD; Maureen Daly, RN; Caitlin Egan, MS; Erica Ferguson, BS, RD; Linda Foss, MPH; Jennifer Gauvin, BS; Don Kieffer, PhD; Lauren Lessard, BS; Deborah Maier, MS; JP Massaro, BS; Tammy Monk, MS; Rob Nicholson, PhD; Erin Patterson, BS; Suzanne Phelan,

PhD; Hollie Raynor, PhD, RD; Douglas Raynor, PhD; Natalie Robinson, MS, RD; Deborah Robles; Jane Tavares, BS

The University of Texas Health Science Center at San Antonio Helen P. Hazuda, PhD[1]; Maria G. Montez, RN, MSHP, CDE[2]; Carlos Lorenzo, MD[3]; Charles F. Coleman, MS, RD; Domingo Granado, RN; Kathy Hathaway, MS, RD; Juan Carlos Isaac, RC, BSN; Nora Ramirez, RN, BSN

VA Puget Sound Health Care System / University of Washington Steven E. Kahn, MB, ChB[1]; Anne Kure, BS[2]; Edward J. Boyko, MD, MPH[3]; Edward Lipkin, MD, PhD[3]; Dace Trence, MD[3]; Subbulaxmi Trikudanathan, MD, MRCP, MMSc[3]; Elaine Tsai, MD[3]; Brenda Montgomery, RN, MS, CDE; Ivy Morgan-Taggart; Jolanta Socha, BS; Lonnese Taylor, RN, BS; Alan Wesley, BA

Southwestern American Indian Center, Phoenix, Arizona and Shiprock, New Mexico William C. Knowler, MD, DrPH[1]; Paula Bolin, RN, MC[2]; Tina Killean, BS[2]; Maria Cassidy-Begay, BSND, RND [2]; Katie Toledo, MS, LPC[2]; Cathy Manus, LPN[3]; Jonathan Krakoff, MD[3]; Jeffrey M. Curtis, MD, MPH[3]; Sara Michaels, MD[3]; Paul Bloomquist, MD[3]; Peter H. Bennett, MB, FRCP[3]; Bernadita Fallis, RN, RHIT, CCS; Diane F. Hollowbreast; Ruby Johnson; Maria Meacham, BSN, RN, CDE; Christina Morris, BA; Julie Nelson, RD; Carol Percy, RN, MS; Patricia Poorthunder; Sandra Sangster; Leigh A. Shovestull, RD, CDE; Miranda Smart; Janelia Smiley; Teddy Thomas, BS

University of Southern California Anne Peters, MD[1]; Siran Ghazarian, MD[2]; Elizabeth Beale, MD[3]; Kati Konersman, RD, CDE; Brenda Quintero-Varela; Edgar Ramirez; Gabriela Rios, RD; Gabriela Rodriguez, MA; Valerie Ruelas MSW, LCSW; Sara Serafin-Dokhan; Martha Walker, RD

Coordinating Center

Wake Forest University Mark A. Espeland, PhD[1]; Judy L. Bahnson, BA, CCRP[3]; Lynne E. Wagenknecht, DrPH[1]; David Reboussin, PhD[3]; W. Jack Rejeski, PhD[3]; Alain G. Bertoni, MD,

MPH[3]; Wei Lang, PhD[3]; David Lefkowitz, MD[3]; Patrick S. Reynolds, MD[3]; Denise Houston, PhD[3]; Mike E. Miller, PhD[3]; Laura D. Baker, PhD[3]; Nicholas Pajewski, PhD[3]; Stephen R. Rapp, PhD[3]; Stephen Kritchevsky, PhD[3]; Haiying Chen, PhD, MM[3]; Valerie Wilson, MD[3]; Delia S. West, PhD[3]; Ron Prineas, MD[3]; Tandaw Samdarshi, MD[3]; Amelia Hodges, BS, CCRP[2]; Karen Wall[2]; Carrie C. Williams, MA, CCRP[2]; Andrea Anderson, MS; Jerry M. Barnes, MA; Tara D. Beckner; Valery S. Effoe, MD, MS; Melanie Franks, BBA; Katie Garcia, MS; Sarah A. Gaussoin, MS; Candace Goode; Michelle Gordon, MS; Lea Harvin, BS; Mary A. Hontz, BA; Don G. Hire, BS; Patricia Hogan, MS; Mark King, BS; Kathy Lane, BS; Rebecca H. Neiberg, MS; Julia T. Rushing, MS; Debbie Steinberg, BS; Jennifer Walker, MS; Michael P. Walkup, MS;

Central Resources Centers

Central Laboratory, Northwest Lipid Metabolism and Diabetes Research Laboratories Santica M. Marcovina, PhD, ScD[1]; Jessica Hurting[2]; John J. Albers, PhD[3], Vinod Gaur, PhD[4]

ECG Reading Center, EPICARE, Wake Forest University School of Medicine

Elsayed Z. Soliman MD, MSc, MS[1]; Charles Campbell [2]; Zhu-Ming Zhang, MD[3]; Mary Barr; Susan Hensley; Julie Hu; Lisa Keasler; Yabing Li, MD

Hall-Foushee Communications, Inc.

Richard Foushee, PhD; Nancy J. Hall, MA

Federal Sponsors

National Institute of Diabetes and Digestive and Kidney Diseases Mary Evans, PhD; Van S. Hubbard, MD, PhD; Susan Z. Yanovski, MD

National Heart, Lung, and Blood Institute Lawton S. Cooper, MD, MPH; Peter Kaufman, PhD, FABMR; Mario Stylianou, PhD

Centers for Disease Control and Prevention Edward W. Gregg, PhD; Ping Zhang, PhD

[1] Principal Investigator

[2] Program Coordinator

[3] Co-Investigator

*deceased

All other LookAHEAD staff are listed alphabetically by site.
